# Opportunities and challenges of supervised machine learning for the classification of motor evoked potentials according to muscles

**DOI:** 10.1186/s12911-023-02276-3

**Published:** 2023-10-02

**Authors:** Jonathan Wermelinger, Qendresa Parduzi, Murat Sariyar, Andreas Raabe, Ulf C. Schneider, Kathleen Seidel

**Affiliations:** 1 Department of Neurosurgery, Inselspital, Bern University Hospital, and University of Bern, Bern, Switzerland; 2 Department of Neurosurgery, Lucerne Cantonal Hospital, Lucerne, Switzerland; 3 School of Engineering and Computer Science, Bern University of Applied Sciences, Biel, Switzerland

**Keywords:** Machine learning, Intraoperative neurophysiological monitoring, Motor evoked potential, Random forest, Time series data

## Abstract

**Background:**

Even for an experienced neurophysiologist, it is challenging to look at a single graph of an unlabeled motor evoked potential (MEP) and identify the corresponding muscle. We demonstrate that supervised machine learning (ML) can successfully perform this task.

**Methods:**

Intraoperative MEP data from supratentorial surgery on 36 patients was included for the classification task with 4 muscles: Extensor digitorum (EXT), abductor pollicis brevis (APB), tibialis anterior (TA) and abductor hallucis (AH). Three different supervised ML classifiers (random forest (RF), k-nearest neighbors (kNN) and logistic regression (LogReg)) were trained and tested on either raw or compressed data. Patient data was classified considering either all 4 muscles simultaneously, 2 muscles within the same extremity (EXT versus APB), or 2 muscles from different extremities (EXT versus TA).

**Results:**

In all cases, RF classifiers performed best and kNN second best. The highest performances were achieved on raw data (4 muscles 83%, EXT versus APB 89%, EXT versus TA 97% accuracy).

**Conclusions:**

Standard ML methods show surprisingly high performance on a classification task with intraoperative MEP signals. This study illustrates the power and challenges of standard ML algorithms when handling intraoperative signals and may lead to intraoperative safety improvements.

**Supplementary Information:**

The online version contains supplementary material available at 10.1186/s12911-023-02276-3.

## Background

Intraoperative neurophysiological monitoring (IOM) has become an integral part of high-risk neurosurgical and orthopedic procedures [1]. Monitoring motor evoked potentials (MEP) is a key tool for assessing the functional integrity of motor pathways during supratentorial, infratentorial and spinal surgeries and predicting motor outcome [2–11]. The parameters that we traditionally extract from MEPs, such as amplitude, motor threshold and morphology, vary considerably even in healthy subjects and therefore make interpretation of the signals challenging [9]. For instance, this lack of clear features to uniquely identify muscle groups can lead to labelling errors resulting in false positive or false negative alarms [12]. In addition, the quality of IOM data is often poor due to the noisy operating-room setting and the influence of numerous environmental factors [10].

The use of machine learning (ML) in medical research and clinical practice has rapidly expanded over recent years. ML been applied in diagnosis and prognosis as well as in classification of diseases [13–15]. Recently, there has been an increased interest in applying ML to IOM data [16]. Among other examples, Holze et al. applied supervised ML to facial surface electromyography (EMG) data to assess facial function [17], Jamaludin et al. used algorithms to predict functional outcomes based on transcranial MEPs [18] and Zha et al. used neural networks to investigate automated classification of free-running EMG waveforms [19]. Presently, Mirallave Pescador et al. propose to use Bayesian Networks to assess evidence in IOM [20].

ML can handle a large amount of data and can support the decision-making process [21]. ML models are generally expected to generate improved results by continuing their learning on additional data. However, the performance of ML algorithms depends critically on the choice of data, its quality (which may be improved by adequate preprocessing) and methods used to prevent bias and overfitting [21, 22].

In this proof-of-concept study, we focused on the classification of MEPs according to the muscles they were recorded from. We opted for this setting instead of a more complex task, such as prediction of postoperative outcome, primarily because the ground truth is not subject to observer bias. This simple task serves as a model to assess opportunities and limitations of different ML paradigms in handling MEP data. In this context, we were interested in how well these ML algorithms perform on completely unprocessed data compared to minimally preprocessed and feature engineered data. This is a first step toward the implementation of ML algorithms for more complex tasks that may help improve safety, for example via automatic alarms in case of mislabeling. Furthermore, using ML to understand MEPs and their features in the muscle identification task could lead to the refinement of MEP warning criteria in the future.

## Materials and methods

This study was approved by the local ethics committee according to Swiss guidelines. All included patients gave their informed written consent for further use and publication of their anonymized data.

### Signal recordings and MEP data

**Fig. 1:**
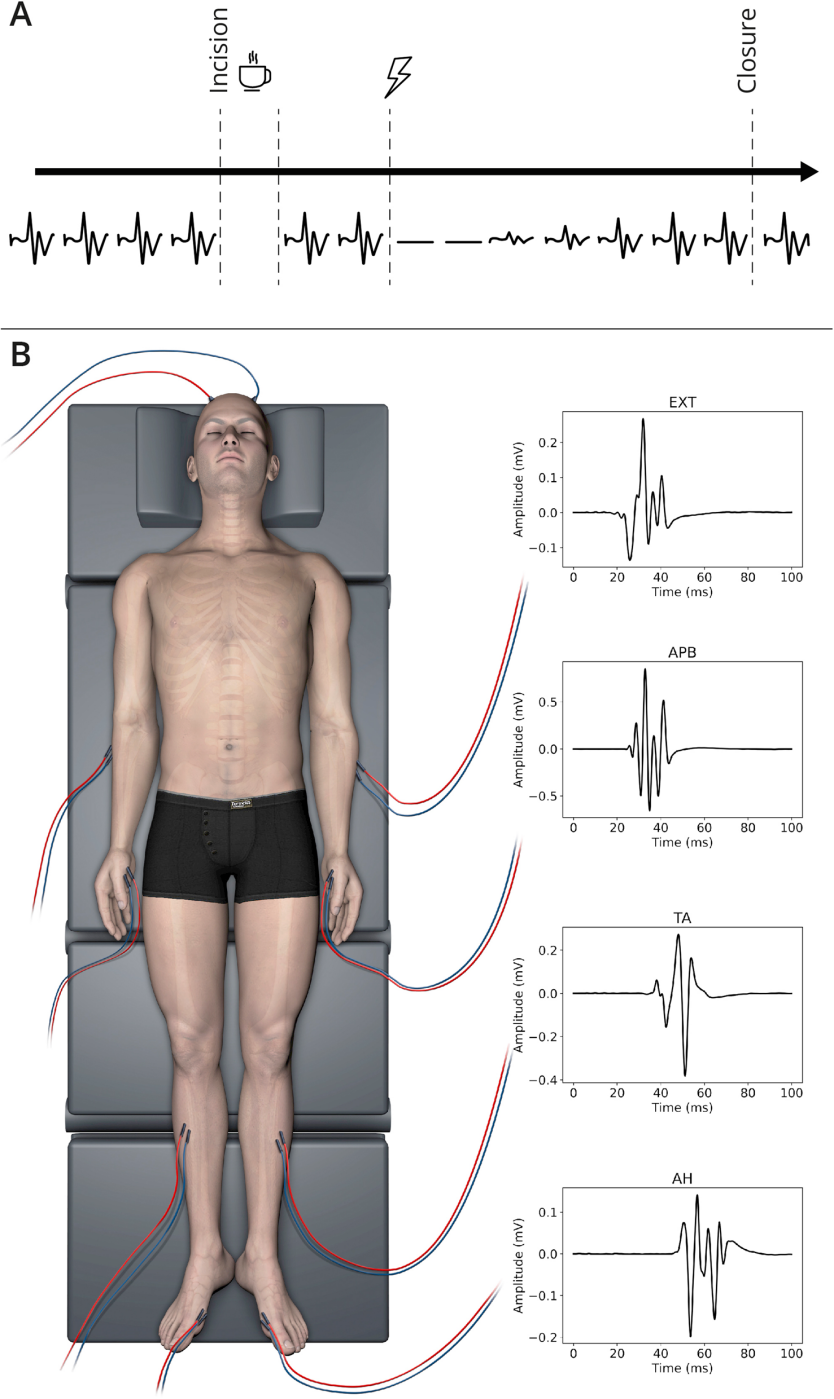
MEP data collection. **A** Timeline of MEP data collection during a surgical operation illustrating that MEPs get recorded irregularly and not continuously. **B** Location of the 4 recorded muscles and sample MEP traces for each muscle. © Inselspital, Bern University Hospital, Dept. of Neurosurgery

MEPs are bioelectrical signals recorded from muscles in response to stimulation of the motor cortex or corticospinal tract [6]. In IOM, MEPs are usually elicited via electric stimulation through corkscrew electrodes at the scalp and recorded via needle electrodes in the muscle belly (Fig. 1). The MEP data used in this study was retrospectively analyzed and stems from IOM recordings during neurosurgical procedures on 36 patients. There were 16 females, 20 males, the mean age at surgery was 59, 35 patients were operated for a brain tumor, one patient for a vascular pathology, 19 patients had no pre-operative motor deficits, 8 had mild and 9 had moderate or severe motor deficits before the surgery. IOM was performed according to a standardized protocol, as previously described [8, 23], at a single center (Inselspital, University Hospital Bern, Switzerland), from 2018 to 2022. The MEPs were recorded with ISIS Systems (Inomed, Emmendingen, Germany). The sampling frequency was 20 kHz and hardware high- and low-pass filters with a cutoff of 30 Hz and 5 kHz, respectively, were used on the machine. We first restricted the recordings to 2000 data points, corresponding to 100 ms windows for each signal. We focused on MEP signals from 4 muscles which are routinely monitored during supratentorial surgeries and were available in all included patients (Fig. 1): Extensor digitorum (EXT), abductor pollucis brevis (APB), tibialis anterior (TA), and abductor hallucis (AH).

The raw data was exported as EDF files from the IOM device and further processed using custom-made Python 3 scripts.

### Exploratory data analysis

To assess the variability of our recorded data, we performed peak detection and explored both amplitude and latency distribution in all patients. The peaks were detected after a cutoff of 17.5 ms to exclude stimulation artifacts and amplitude was measured as the absolute difference of the minimum to the maximum of the signal.

The onset latency was also detected starting at a cutoff of 17.5 ms and was defined as the time point when the signal crosses the mean of the last 20 ms of the window (for which it was expected that there was no signal) plus or minus 2 times the standard deviation of the signal trace.

### Machine learning pipeline

**Fig. 2:**
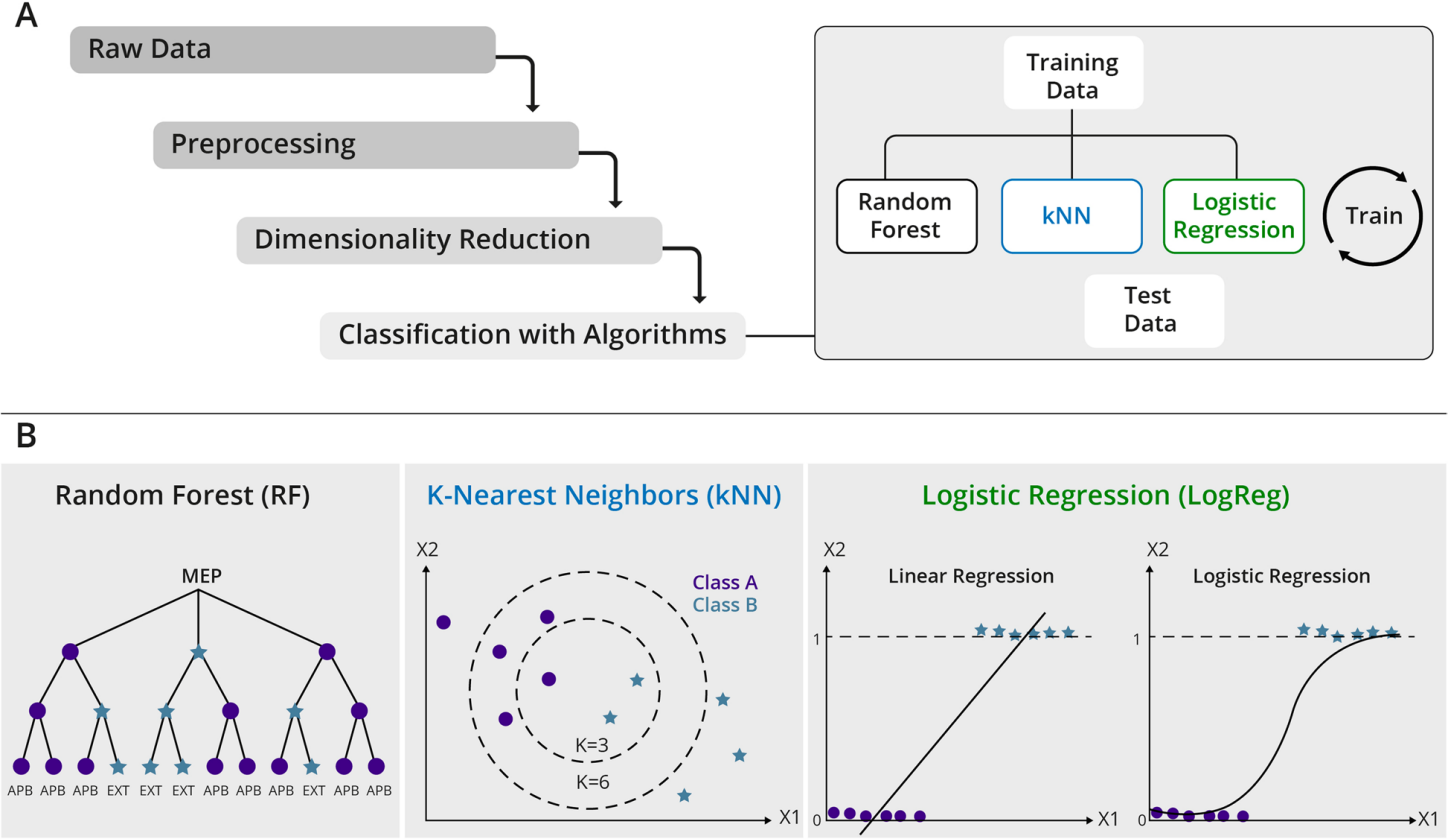
Machine learning pipeline. **A** The raw data is preprocessed (preselected and augmented) and then either directly used to train and test the 3 supervised ML algorithms, or compressed via dimensionality reduction methods (PCA or feature extraction). **B** Illustrations of the 3 algorithms applied to classify the data. Random forests use a majority vote of decision trees, k-nearest neighbors classify the data according to some distance metric, and logistic regression is a statistical method estimating the probability of a data point belonging to a certain class

We used a standard approach to implement a ML pipeline (Fig. 2A). Python 3 [24] scripts were written to carry out each of the steps, making extensive use of the *scikt-learn* package [25]. When algorithms or methods are mentioned this refers to ML algorithms or methods. We implemented 3 standard supervised learning algorithms for this study (see Fig. 2B) [21]:

- *Random forest (RF)* [26]: an ensemble learning method making use of multiple decision trees for supervised classification
- *K-nearest neighbor (kNN)* [27]: a supervised learning classifier, which uses proximity to make classifications or predictions about the grouping of an individual data point
- *Logistic regression (LogReg)* [28]: a statistical method that estimates the parameters of a logistic model to classify data.

### Preselection

A Python function was written to detect peaks with a prominence of more than 2 standard deviations, starting after 17.5 ms. If at least one and no more than 10 peaks were detected, the signal was assumed to contain an MEP. During preselection, the first 350 data points (or 17.5 ms) of the signal were removed, in order to get rid of the train of five stimulation artifacts. Therefore, the final dimension of each MEP used for the ML application was 1650. Furthermore, all the MEPs were normalized by dividing each trace by the absolute maximum value of each individual patient’s highest peak MEP value.

### Splitting and data augmentation

The data from 28 patients was used for the training dataset, while the data of the remaining 8 patients was used for the test dataset (all randomly selected). After preselection, this corresponded to approximately an 80:20 split [21]. In order to cope with the imbalance in the training dataset (more arm MEPs than leg MEPs), we used the synthetic minority oversampling technique (SMOTE) [29]).

### Tuning the hyperparameters

All of the ML algorithms used depend on parameters that must be chosen and optimized in order to obtain the best results. In our case, this was carried out using the built-in *GridSearchCV* function of the *model_selection* module of *scikit-learn* [21, 25]. We tuned these parameters on all the different types of training data and for all the different algorithms (see Supplementary material - Additional file 1).

### Dimensionality reduction

Dimensionality reduction methods were used to reduce the time needed for the training of the algorithms on the raw data [30] and to compare the performances. In this study, the following standard methods were used:

- Principal component analysis (PCA): a technique which linearly transforms the data into a new coordinate system that captures (most of) the variation of the data with fewer dimensions [31].
- A simple feature extraction (FE) was carried out using a custom-made Python function to extract onset latency (see Sect. "Exploratory data analysis".), peak latency (i.e. latency of the first peak), end of signal (defined as the onset latency of the inverse of the signal), maximum, minimum, area under the curve (AUC), and number of peaks.

### Training the classifiers

To train and test the 3 supervised ML algorithms the data was separated in the following ways.

First, we grouped the data according to muscle comparison paradigms:

- Four muscles simultaneously (*APB versus EXT versus TA versus AH*)
- Within upper extremity comparison (*EXT versus APB*)
- Across extremities comparison (*EXT versus TA*).

We explained in Sect. "Splitting and data augmentation". how we obtained the training and test data sets for the 4-muscle comparison. To obtain the data sets for the within upper and across extremity comparison, we simply dropped the appropriate rows in X_train_ and X_test_ with the corresponding labels from y_train_ and y_test_.

Then, for each of the input data matrices, the data was again separated after applying one of the following strategies for compressing data:

- Raw, unprocessed data (dimensions per signal: 1650)
- Data reduced by PCA (reduced to capture 95% variability of data, dimensions per signal: 20–40)
- Feature extracted data (dimensions per signal: 7, see Sect. "Dimensionality reduction".)

The 3 different algorithms, with the parameters specified in the previous section, were then trained on all of these different training datasets ($3\times 3=9$ in total) and their performance evaluated first through a cross validation (CV) with 10 folds and finally on the test dataset.

### Assessing performance

We assessed the performance of each algorithm in each scenario based on the following 3 scores [21]:

- *Accuracy*: The percentage given by the number of correct classifications divided by the total number of samples in the test dataset.
- *ROC AUC*: Receiver operating characteristic area under the curve plotting the true positive rate against the false positive rate.
- *F1*: A performance metric that combines the precision (positive predictive value) and recall (sensitivity) scores of a model. The formula is:
  $$F1= \frac{2}{\frac{1}{Recall}+\frac{1}{Precision}}=\frac{2TP}{2TP+FP+FN}$$
  where TP stands for true positive, FP for false positive and FN for false negative. In the case of the 4-muscle comparison, which is a multiclass problem, we used the so-called ‘macro’ weighting, which determines the F1 score for each label and computes their unweighted mean.

For each algorithm in each paradigm, the final scores are the performance on the test dataset.

### Neurophysiologist task sheets

A questionnaire (see Supplementary material: Additional file 2) with 20 labeled MEPs (5 MEPs for each of the 4 muscles, from a single patient) on the front and 19 unlabeled MEPs (5 EXT, 5 APB, 5 TA and 4 AH) on the back was handed to 30 experienced neurophysiologists. They were instructed to look at the front of the sheet to “train” and learn the MEP properties, and then to turn the paper over and classify the MEPs presented into one of the 4 muscle groups (EXT, APB, TA, AH). The results were collected and the overall accuracy, as well as the confusion matrix, were determined.

## Results

### MEP data

**Fig. 3:**
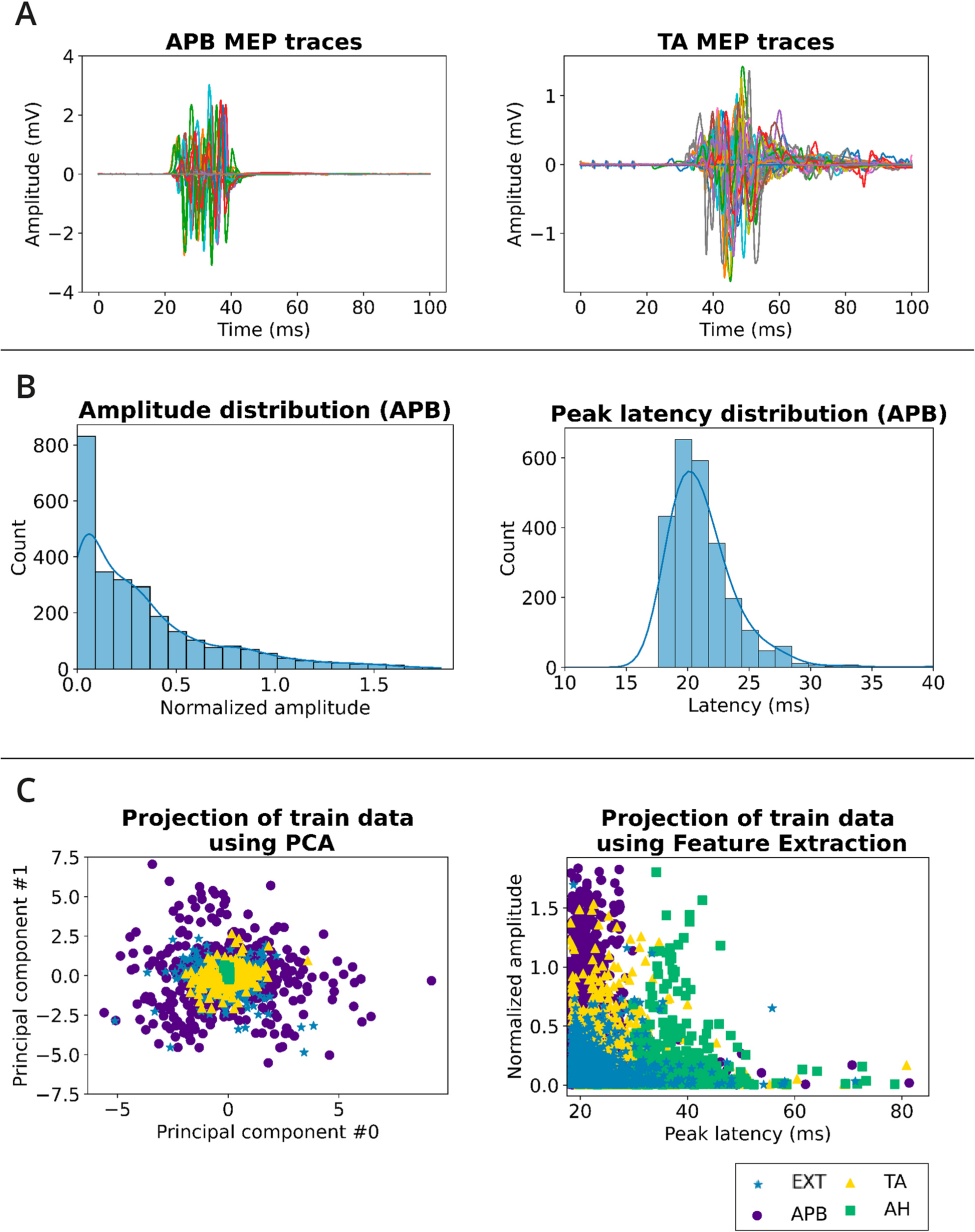
MEP properties to illustrate the high variability of intrinsic MEP signal features. **A** MEP traces recorded during an entire surgery plotted on top of each other for 2 different patients and muscles (left: all APB MEPs of one patient, right: all TA MEPs of a second patient). Different colors indicate different MEP recordings. **B** Distribution of onset latencies and amplitude distributions of the APB muscle for all patients. **C** Dimensionality reduction of test data via principal component analysis (left) and feature extraction (right). For the PCA plot, the data is displayed along the 2 components of highest data variability, whereas on the right, 2 intuitive features were selected for the x- and y-axis. Each dot represents an MEP signal

The data from 36 surgeries yielded a total of 4038 EXT, 4911 APB, 4821 TA and 4038 AH MEP recordings. The preselection function identified 3016 EXT, 3496 APB, 1451 TA and 835 AH traces containing an MEP. After the splitting (see Sect. "Splitting and data augmentation"), the training dataset contained 2274 EXT, 2665 APB, 1221 TA and 710 AH MEPs, while the test dataset contained 742 EXT, 831 APB, 230 TA and 125 AH MEPs. Average normalized peak amplitudes ranged between 0.09 and 0.34, average latencies of muscles were between 17.5 ms and 31.0 ms, and median number of peaks was either 1 or 2, with variability in the standard deviations (see Table 1). During a single surgery, the signal traces can vary significantly as illustrated for 2 patients with different muscle recordings (Fig. 3A) and MEP feature distributions over all patients (Fig. 3B).

**Table 1:** Summary statistics of the recorded MEP properties

Muscle	Normalized Amplitude	Peak latency (ms)	# Peaks	Normalized AUC
**EXT**	0.09 ± 0.12	17.53 ± 10.14	1 ± 1.5	0.4 ± 0.6
**APB**	0.34 ± 0.36	20.01 ± 6.38	2 ± 1.1	1.02 ± 1.29
**TA**	0.20 ± 0.25	24.69 ± 9.36	2 ± 1.5	0.84 ± 1.13
**AH**	0.11 ± 0.23	31.03 ± 15.94	2 ± 1.8	2.0 ± 1.83

Mean ± standard deviation over all preselected MEPs, except for the number of peaks, which indicates median ± standard deviation

### Classification

The results of the hyperparameter tuning can be found in [Supplementary material: Additional file 1].

#### Four muscles

**Fig. 4:**
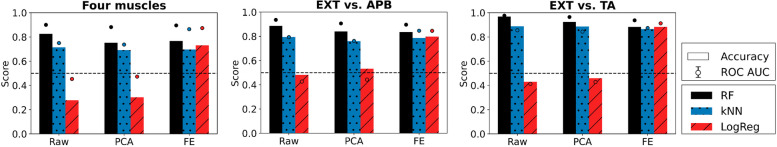
Performances of the classification methods. Depicted are accuracy (bars) and ROC AUC (dots) values for the color-coded algorithms for all three paradigms. The scores are the result of evaluating the methods on the single test dataset (MEP data of 8 patients). The RF classifier performed best overall and on the raw data in particular. The kNN classifier performed second best overall. Note that the LogReg performed poorly on raw and PCA data, but well on feature extracted data (in all paradigms)

**Fig. 5:**
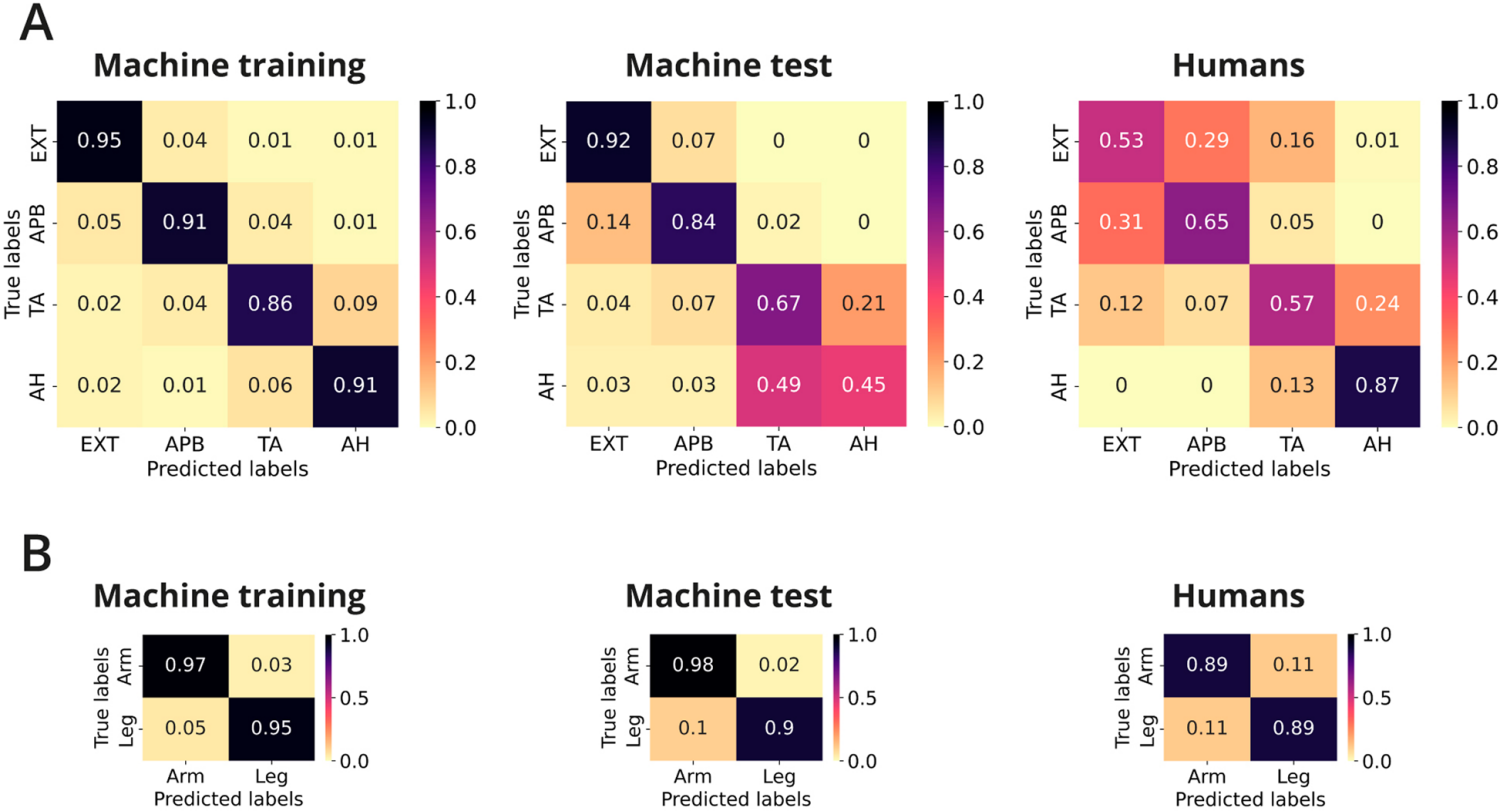
Confusion matrix of the best machine learning algorithm compared to human classification. **A** Four-muscle classification performance (normalized by rows and rounded). Depicted is the confusion matrix of the RF on raw data for the cross validation during training (left) and the test (middle), compared to the results of the human classification (right). The RF can distinguish all 4 muscles extremely well during training, but has more difficulties classifying lower limb muscles in the test dataset. Due to the reliance on latency as the main distinguishing criterion, the neurophysiologists can confidently differentiate between upper and lower limbs, but have poorer performance on individual muscles. **B** Limb classification performance (normalized by rows and rounded). The scores from the matrices in (**A**) are summed across limbs. This shows the good differentiation of upper versus lower extremities by both the ML algorithm and the neurophysiologists

The MEPs were first classified in their raw form, then using PCA and a representation of their main features (FE). The RF and kNN classifiers performed best and second best respectively (Fig. 4 and Table 2) and consistently above chance level. For these best performing algorithms, the raw data setting was optimal (accuracy: RF 83%, kNN 71%). In both cases however, the F1 score (RF 72%, kNN 64%) together with the confusion matrix (Fig. 5) reveal disparities in classification performances with certain muscles. Feature engineering (FE) yielded a considerable improvement in the LogReg classifier (raw 28%, feature engineering 73% accuracy). In the cases where accuracy was high, the ROC AUC values were high as well, corroborating the good class separability.

**Table 2:** Test performance scores

		**Raw**			**PCA**			**FE**		
		**Acc**	**F1**	**ROC AUC**	**Acc**	**F1**	**ROC AUC**	**Acc**	**F1**	**ROC AUC**
**Four muscles**	**RF**	**0.83**	0.72	0.9	0.75	0.67	0.88	0.77	0.71	0.9
	**kNN**	0.71	0.64	0.75	0.69	0.6	0.74	0.7	0.66	0.86
	**LogReg**	0.28	0.24	0.45	0.3	0.26	0.47	0.73	0.67	0.87
**EXT vs. APB**	**RF**	**0.89**	0.88	0.94	0.84	0.84	0.91	0.83	0.83	0.9
	**kNN**	0.79	0.79	0.79	0.76	0.76	0.76	0.79	0.79	0.85
	**LogReg**	0.48	0.47	0.85	0.53	0.5	0.44	0.8	0.8	0.85
**EXT vs. TA**	**RF**	**0.97**	0.95	0.98	0.92	0.9	0.96	0.88	0.84	0.94
	**kNN**	0.89	0.85	0.85	0.89	0.84	0.84	0.87	0.82	0.87
	**LogReg**	0.43	0.4	0.41	0.46	0.42	0.43	0.88	0.85	0.91

Bold: best performance for each paradigm. Accuracy (Acc) reflects the percentage of correctly assigned labels. ROC AUC is the area under the curve plotting the true positive against the false positive rate. A high ROC AUC means that the model is good at distinguishing between the positive and negative classes. The F-score (F1), macro weighted in case of multiclass classification, is the harmonic average of precision (also known as positive predictive value) and recall (also known as sensitivity). A good F1 can only be achieved if both precision and recall are high

#### Two muscles

To further assess the classification abilities of the algorithms, we compared their performances in 2 further settings: one where 2 muscles within the upper extremity were classified (*EXT versus APB*), and the other was a classification task for separating a muscle from the upper and one from the lower extremities (*EXT versus TA*). In both settings, RF classifier again performed best with kNN being second best in the raw data setting (*EXT versus APB:* RF 89%, kNN 79%; *EXT versus TA*: RF 97%, kNN 89%), surpassing the performance of the 4-muscle scenario overall but also showing a better performance in the opposing limb scenario. Remarkably, the performance of the LogReg classifier in combination with feature engineering achieved performances comparable to the best performing algorithms (*EXT versus APB* 80% and *EXT versus TA* 88%), showing the importance of adequate data structuring for specific algorithms.

### Benchmarking human performance

To compare misclassifications by the algorithm with those from the human assessment, we compared the best performing algorithm (RF classifier on raw MEP traces across 30 patients) with the performance of 30 neurophysiologists classifying a selection of 19 MEPs from one patient. Although this cannot be seen as a direct comparison of performance, it illustrates the decision-making process (Fig. 5A). The RF classifier shows very high values in the training set along the diagonal axis, illustrating the precision with which it can classify the muscles. However, the values are lower for the lower extremity muscles where there is a higher misclassification of the AH muscle, a substantial amount being predicted as TA. This is confirmed by the relatively low accuracy on the test data set (Fig. 5A). At first glance the human decision-making matrix seems more scattered; however, the same trend is visible along the diagonal axis. The experts achieved an accuracy above 50%, which was highest in the AH (87%). However, a closer look at the human confusion matrix shows considerably more contextual information. As shown in Fig. 5B, the human experts catch up with ML performance, achieving 89% accuracy when it comes to identifying which limb the MEPs belong to.

## Discussion

With our proof-of-concept study, we demonstrate that classical ML algorithms are able to classify MEPs according to muscle groups with high accuracy. This could improve IOM safety by signaling mislabeling of muscles, which can have detrimental consequences by harming the patient. Furthermore, it is a first step toward the implementation of ML algorithms for more complex tasks that may help improve MEP warning criteria. Thus, ML could help to overcome some of the intrinsic difficulties of intraoperative neurophysiological data. The opportunities and limitations are discussed below based on our exemplary model of MEP classification.

### Data quality and class imbalance

Crucial aspects that determine the performance of ML are the quality of the data and the adequacy of labeling [32]. In our case of MEP classification, the labeling is not subject to observer bias, unlike, for instance, the clinical assessment of motor performance. Nevertheless, the training data needs to be thoroughly checked for outliers, noise and, ultimately, for a balanced representation of each class. After removing noisy data from our dataset, we were faced with a class imbalance (see Sect. "MEP data"). There were fewer lower extremity MEPs than upper extremity MEPs, which is likely due to the way stimulation for MEPs is commonly carried out during supratentorial surgery. Unless lower extremity muscles are at risk, a threshold current is applied to elicit a crucial number of upper extremity muscles [9], which leads to many more ‘blanks’ in the lower extremity data. These blanks were subsequently removed in data preprocessing. Furthermore, due to the placement of cortical strip electrodes on the hand motor cortex [8], direct cortical stimulation generates a bias toward an increased collection of upper extremity MEPs. To compensate, we used SMOTE [29] for data augmentation (see Sect. "Splitting and data augmentation"). This, of course, is not the same as having additional MEP recordings, but constitutes a common approach to balance the different classes, which has been successfully used in the past. However, as demonstrated in the confusion matrix (Fig. 5), the imbalance is visible as a lower accuracy in the classification of lower extremity classes. This effect has to be taken into account when interpreting ML results. Ignoring data quality problems could lead to the assumption that the classification of lower extremity signals is more difficult based on intrinsic properties, which in the case of poor, noisy or imbalanced data might be premature [33].

### Importance of model and parameter choices

We implemented 3 ML algorithms with the same task of classifying MEPs (Fig. 2). These algorithms have different mechanisms for learning class representations. While certain algorithms may be chosen because they perform better on certain types of data, at least the same emphasis should be put on the choice of model parameters to achieve these performances. It is important to understand the source of prediction errors as well as the distinction between *bias*, *variance* and *noise* [34].

The *bias* of a model is the deviation of the outcome from what we expect. For example, if we had an infinite amount of training data from all muscles, but we set the model to focus on the amplitude (through preprocessing or feature selection). In this way, we would systematically achieve better results for upper extremity classification, because they happen to have bigger amplitudes. This is an inherent problem of the model choice.

The contribution of *variance* to the prediction error is a measure of how much a classifier changes with a given training set (i.e., how much does it overspecialize on the data it has seen and how hard is it to generalize to new data). Learning this type of variance in the data is also known as overfitting [35]. *Noise* is limited to intrinsic noisiness of the data due to the measurement process (recording, amplification etc.), and cannot be reduced by algorithm parameter choices.

There are various approaches to address the *bias–variance* tradeoff and we chose to do cross-validations to select parameters (see Sect. "Tuning the hyperparameters"). Many classifiers have a parameter that directly handles this tradeoff. For example, the choice of number of neighbors in the kNN algorithm determines how much the classifier will generalize. If we choose a large number of neighbors to compare to, the kNN model will always get the general trend right (arm versus leg), but it will fail to give the precise muscle. However, if we set the kNN to focus only on the next neighbor (k = 1), the algorithm would put more emphasis on the variance of the data and assume there is meaningful content hidden in that. This would likely throw the algorithm off course when presented with new data and lead to overfitting.

Another option for reducing the variance of the model is to choose simpler, lower dimensional representations of the data, which minimizes the focus on the variance and captures essential elements of the data instead. This is the goal of PCA compression and feature engineering (see Sect. "Dimensionality reduction" and Fig. 3C, where in feature engineering we represent the data with latencies, amplitudes and other shape parameters of the MEPs). With adequate data representations that suit the algorithm’s learning method, performance can sometimes be dramatically improved, as exemplified in our case by the LogReg algorithm (Fig. 4). We assume that more extensive feature engineering, for instance by weighting different aspects of the data, might further improve performance.

Ensemble learning, where multiple learning algorithms are combined to obtain more robust and accurate predictions [36], is also used to reduce the impact of variance. The RF algorithm is one example of an ensemble learning method that uses a bagging (**b**ootstrap **agg**regati**ng** method) to avoid overfitting. It takes the average (a majority vote) of different decision-tree models and achieves best performances this way (as observed in our case).

### Interpretation of machine learning results

Interpretation of ML results depends on an understanding of the processing pipeline and the drawbacks of the different algorithms. Although the classification task we chose is relatively straightforward, it is perfect to exemplify the pitfalls of ML in general. In Sects. "Data quality and class imbalance" and "Importance of model and parameter choices", we addressed problems of class imbalance and bias-variance tradeoff. Special attention is also needed in the choice of metrics (or scores) used for performance assessment. Whereas accuracy is an intuitive scoring method, useful for assessing how well the classification is working, it does not allow for an adequate evaluation of performance on imbalanced classes. Thus, if our dataset comprised mostly upper extremity muscles, a bad model will predict this majority class correctly and thus reach high accuracy, but might always be wrong in the lower extremity examples. Our setting is relatively balanced compared to clinical settings, where intraoperative IOM alterations leading to permanent postoperative motor deficits in the patients are relatively rare but need to be diligently avoided [10].

Furthermore, we tried to remedy the class imbalance of our data by augmenting the training dataset. If we only considered accuracy and not the confusion matrix, we would have missed the remaining influence of the imbalance. This is why it is important to take into account additional performance metrics and scores, such as the confusion matrix, ROC AUC and F1 scores (see Sect. "Assessing performance" and [33, 37]). In our case, the ROC AUC scores of the RF and kNN models were high, but F1 scores dropped at times (Table 2) due to the class imbalance, therefore highlighting the importance of referring to various scores to asses ML performance.

Similarly, it is misleading to compare performances of a 2-muscle paradigm with a 4-muscle paradigm, since binary classifications are generally much easier to solve. Many of the standard ML algorithms were designed to deal with a binary classification and were only later adapted to multiclass settings [38]. Thus, higher performance scores in a 2-muscle compared to a 4-muscle setting are quite meaningless. Comparing the 2-muscle tasks with each other, we observed that they performed similarly well, with a better performance in the classification of upper versus lower extremity. This might simply be due to the data imbalance. This can be seen in the confusion matrix (Fig. 5A), where across the diagonal the algorithm misclassifies leg muscles more often than arm muscles.

Neurophysiologists seemed to classify the extremities more consistently, which likely follows from their assumption that all classes were equally represented (which is true). It would be interesting to investigate whether the discrimination between extremities would be as robust if the neurophysiologists were presented with an imbalanced test. Further, due to their background training and education, it is very likely that neurophysiologists put more emphasis on latency, which makes it easier to differentiate between upper and lower extremities than within the same extremity.

Caution is also needed when comparing the results from raw data with the reduced data representation (PCA and FE). The dataset is large enough to implement basic ML methods, but too small to avoid the effects of variance learning. Slightly higher accuracies and ROC AUC performances were achieved in the raw data setting compared to the compressed methods throughout the 3 paradigms.

As mentioned above, feature engineering can certainly be optimized, which would improve performances and understanding of the classification algorithms [39]. Whether this would lead them to surpass the raw data setting is an open question. In any case, this means that the results of this study should not be considered the ultimate benchmark for the compression methods. Another reason for the better performance of the algorithms on raw data could be that we were missing one or more components when compressing the data.

In summary, interpretation of these supervised ML results should be strictly tied to what might be contributing to these results in terms of data properties, rather than trying to attribute results to some type of concept learning.

### Comparing ML results to human decision making

The rise of ML has naturally raised the question of the extent to which human intervention is still necessary, where it can be complemented, and where it could potentially be replaced. IOM operates in a very intricate setting in which many factors can affect the change in signals (anesthetic regimen, electrical noise, neurophysiological stimulation paradigms, surgical intervention, tumor pathology, biological factors of the patient, staff involved, etc.). Moreover, quantification of postoperative clinical and surgical outcome is difficult, leading to unclear labeling. This is a very different situation compared to our simple classification problem. Experienced neurophysiologists show an exceptional ability to contextualize and interpret these difficult intraoperative scenarios to limit false alarms. Indications of this contextual learning can be found in the confusion matrix. Although precision in predicting the exact muscle is lacking (Fig. 5A), the neurophysiologists can confidently assign the correct extremity (Fig. 5B) due to the latency difference. ML has great potential to offer support on more precise questions. For example, it could help improve warning criteria by detecting more subtle changes in MEPs (instead of the current 50%-drop criterion) or certain time series patterns that correlate with postoperative outcome.

### Potential clinical applications

Even if it might not be evident at first glance, we would like to highlight that these preliminary results could have significant clinical impact. During the set-up of IOM, more than 20 needles have to be placed in the correct muscles of the patient, while the connected cables need to be labeled correctly and plugged in the corresponding channel of the EMG system. In case of labeling errors, MEP alterations during the surgery might either be misinterpreted or missed altogether, resulting in false positive or false negative alarms. Those events have been reported and even overlooked during publication in a case report [40]. According to Yingling [12], “the examination of the data indicates that the recording leads from the upper and lower extremities were inadvertently reversed during setup, and the MEP recordings from the lower extremities (mislabeled as upper) were in fact lost early in the procedure. This loss, which would have normally triggered an alert and corrective action, went unnoticed by the authors, with the tragic outcome of postoperative paralysis." This grave mistake might have been avoided if an automatic alarm had been provided by the IOM system.

In the end, the ultimate future goal is to provide a basis to improve warning criteria in MEP monitoring. Various alarm criteria, such as signal loss and amplitude reduction, have been reliably correlated to postoperative motor outcome of the patients [2, 5, 7–11]. However, to date only a limited number of warning criteria have been analyzed and implemented [10]. As indicated above, the traditional MEP parameters vary considerably within and across patients. This may affect MEP monitoring of individual patients during surgery as well as limit generalizability for different patients and monitoring procedures [6]. A systematic and exhaustive feature engineering with and without the help of ML algorithms might lead to a better understanding of the important properties of MEPs. Indeed, our results raise the question of whether the ML algorithms detect neurophysiological markers which have not been considered until now in traditional clinical neurophysiology. There might be additional intrinsic features in MEP traces of different muscles, even within the same extremity (EXT vs APB, Figs. 4 and 5). Further analysis is needed to understand the classification process.

### What is needed to (try to) successfully implement ML in IOM

When faced with good and plentiful data, ML can lead to astonishing results, as exemplified by AlphaGo [41], AlphaZero [42] and many more. In most of these cases however, the “rules” and outcomes are well known, the data is thoroughly labeled, and the quantity of data available is enormous. The relative scarcity, complexity and variability of IOM data and the difficulty of quantifying outcomes of surgeries still renders the implementation of ML in IOM an ambitious enterprise. This is why we thought it important to break down this problem into smaller, more achievable steps. This is similar to how ML algorithms have traditionally been tested and compared on the MNIST dataset [43], made up of labeled pictures of numbers 0 through 9, before trying to implement them in more complex situations.

To tackle the abovementioned challenges, the following should be considered in future work:

- Data quality: high quality recordings, awareness of inherent variability
- Labeling: standardized protocols, clear labeling rules [44]. This should also include tracking sources of variance during the surgical procedure (e.g., expected and unexpected noises, such as cautery, drill or anesthesia processes) and bias of data collection (only upper extremity MEPs for threshold reasons)
- Adequate quantification of the outcome: defined and standardized outcome scores at defined postoperative time points [10] and outcome scores for the ML task, to limit an interpretation bias
- More data [45]: pooled data from multiple centers
- Understanding data: exploratory data analysis to find out how the data is distributed, analyzing imbalances of the features as well as the labels, and extraction of meaningful information to feed into ML, in particular to address to some extent the “black box” of ML [46].

Even beyond potential ML applications, meeting such requirements would constitute good practice for data scientific purposes in IOM (compare with [47]).

## Limitations

In this study, we kept the signal preprocessing to a minimum. Cleaning the data (for example by applying filters), expanding the feature engineering, improving preselection, and applying other classification methods could lead to better performances. In particular, it would also be interesting to compare the performance of the ML algorithms on the raw data to the performance on the same data filtered with the standard filters used on IOM machines during surgery (i.e. with a low-pass filter of 10—100 Hz and high-pass filter of 1.5—3 kHz), to see whether the filtering is removing some important properties of the signals. We only tried one method to deal with the data imbalance, but many others could be tested. Even though we had a reasonable amount of data, current ML algorithms need a large amount of data to reach their full potential [45]. We only applied standard ML algorithms and did not try deep learning methods. Our next step will be to implement neural networks together with various sorts of preprocessing and feature engineering strategies to evaluate their performance and compare them to the standard ML algorithms used in this study.

## Conclusion

This proof-of-concept study may serve as a model to assess opportunities and limitations of different ML paradigms in handling MEP data. We demonstrated that applying various classification methods to MEP data is feasible. Further, we have shown that different combinations of data processing, algorithm training and paradigms robustly classify MEP signals according to their muscle with very high accuracy. However, the robustness of our results should be investigated with larger datasets to gain a more representative understanding of their performances. In addition, systematic and exhaustive feature engineering with and without the help of ML algorithms might lead to a better understanding of the important properties of MEPs.

### Supplementary Information

**Additional file 1.** 

**Additional file 2.** 

## Data Availability

The datasets generated and/or analyzed during the current study are not publicly available due to patient data privacy reasons but are available from the corresponding author on reasonable request.

## Abbreviations

AH: Abductor hallucis
APB: Abductor pollicis brevis
AUC: Area under the curve
CV: Cross validation
EMG: Electromyography
EXT: Extensor digitorum
FE: Feature extraction
IOM: Intraoperative neurophysiological monitoring
MEP: Motor evoked potential
ML: Machine learning
PCA: Principal component analysis
RF: Random forest
ROC: Receiver operating characteristic
SMOTE: Synthetic minority oversampling technique
TA: Tibialis anterior
