# Supplementary material for: Opportunities and challenges of supervised machine learning for the classification of motor evoked potentials according to muscles
Source: BMC Med Inform Decis Mak. 2023 Oct 2;23:198. doi: 10.1186/s12911-023-02276-3 (PMC10544622; doi:10.1186/s12911-023-02276-3)
Supplement: Supplementary file 1 — Additional file 1. [file 12911_2023_2276_MOESM1_ESM.pdf]

Supplementary material: Hyperparameter tuning

The parameters used with the *scikit-learn.model_selection* function *GridSearchCV* were the following.

RF:

n_estimators = [200,500,1000],

min_samples_split = [2,16,64],

random_state = [42],

max_samples = [0.6,0.8],

kNN:

n_neighbors = [1,3,5,7],

weights = ['uniform', 'distance'],

metric = ['cityblock','cosine','l1','l2']

LogReg:

penalty = ['l1', 'l2', 'elasticnet'],

C = [0.1,0.5,1.0],

random_state = [42],

multi_class = ['auto','ovr','multinomial']

The following were the ideal parameters determined by the gridsearch, depending on the algorithm, the type of data and the muscle comparison.

RF_Raw_4

{'RF__max_samples': 0.8, 'RF__min_samples_split': 2, 'RF__n_estimators': 500, 'RF__random_state': 42}

RF_Raw_ext_apb

{'RF__max_samples': 0.8, 'RF__min_samples_split': 2, 'RF__n_estimators': 500, 'RF__random_state': 42}

RF_FE_4

{'RF__max_samples': 0.8, 'RF__min_samples_split': 2, 'RF__n_estimators': 200, 'RF__random_state': 42}

RF_PCA_4

{'RF__max_samples': 0.6, 'RF__min_samples_split': 2, 'RF__n_estimators': 1000, 'RF__random_state': 42}

---

kNN_Raw_4

{'kNN__metric': 'l1', 'kNN__n_neighbors': 1, 'kNN__weights': 'distance'}

---

kNN_FE_4

{'kNN__metric': 'l1', 'kNN__n_neighbors': 5, 'kNN__weights': 'distance'}

---

kNN_PCA_4

{'kNN__metric': 'cityblock', 'kNN__n_neighbors': 1, 'kNN__weights': 'distance'}

LogReg_Raw_4

{'LogReg__C': 1.0, 'LogReg__multi_class': 'auto', 'LogReg__penalty': 'l2', 'LogReg__random_state': 42}

LogReg_FE_4

{'LogReg__C': 1.0, 'LogReg__multi_class': ' multinomial', 'LogReg__penalty': 'l2', 'LogReg__random_state': 42}

LogReg_PCA_4

{'LogReg__C': 1.0, 'LogReg__multi_class': 'ovr', 'LogReg__penalty': 'l2', 'LogReg__random_state': 42}

RF_FE_ext_apb

{'RF__max_samples': 0.8, 'RF__min_samples_split': 2, 'RF__n_estimators': 500, 'RF__random_state': 42}

RF_PCA_ext_apb

{'RF__max_samples': 0.8, 'RF__min_samples_split': 2, 'RF__n_estimators': 1000, 'RF__random_state': 42}

---

kNN_Raw_ext_apb

{'kNN__metric': 'cityblock', 'kNN__n_neighbors': 1, 'kNN__weights': 'uniform'}

---

kNN_FE_ext_apb

{'kNN__metric': 'l1', 'kNN__n_neighbors': 7, 'kNN__weights': 'distance'}

---

kNN_PCA_ext_apb

{'kNN__metric': 'cityblock', 'kNN__n_neighbors': 1, 'kNN__weights': 'uniform'}

LogReg_Raw_ext_apb

{'LogReg__C': 1.0, 'LogReg__multi_class': 'multinomial', 'LogReg__penalty': 'l2', 'LogReg__random_state': 42}

LogReg_FE_ext_apb

{'LogReg__C': 1.0, 'LogReg__multi_class': 'multinomial', 'LogReg__penalty': 'l2', 'LogReg__random_state': 42}

LogReg_PCA_ext_apb

{'LogReg__C': 0.5, 'LogReg__multi_class': 'multinomial', 'LogReg__penalty': 'l2', 'LogReg__random_state': 42}

RF_Raw_ext_ta

{'RF__max_samples': 0.8, 'RF__min_samples_split': 2, 'RF__n_estimators': 1000, 'RF__random_state': 42}

RF_FE_ext_ta

{'RF__max_samples': 0.8, 'RF__min_samples_split': 2, 'RF__n_estimators': 500, 'RF__random_state': 42}

RF_PCA_ext_ta

{'RF__max_samples': 0.8, 'RF__min_samples_split': 2, 'RF__n_estimators': 1000, 'RF__random_state': 42}

---

kNN_Raw_ext_ta

{'kNN__metric': 'l1', 'kNN__n_neighbors': 1, 'kNN__weights': 'distance'}

---

kNN_FE_ext_ta

{'kNN__metric': 'cityblock', 'kNN__n_neighbors': 3, 'kNN__weights': 'uniform'}

---

kNN_PCA_ext_ta

{'kNN__metric': 'cosine', 'kNN__n_neighbors': 1, 'kNN__weights': 'distance'}

LogReg_Raw_ext_ta

{'LogReg__C': 1.0, 'LogReg__multi_class': 'auto', 'LogReg__penalty': 'l2', 'LogReg__random_state': 42}

LogReg_FE_ext_ta

{'LogReg__C': 1.0, 'LogReg__multi_class': 'multinomial', 'LogReg__penalty': 'l2', 'LogReg__random_state': 42}

LogReg_PCA_ext_ta

{'LogReg__C': 0.1, 'LogReg__multi_class': 'auto', 'LogReg__penalty': 'l2', 'LogReg__random_state': 42}
