# Supplementary material for: Opportunities and challenges of supervised machine learning for the classification of motor evoked potentials according to muscles
Source: BMC Med Inform Decis Mak. 2023 Oct 2;23:198. doi: 10.1186/s12911-023-02276-3 (PMC10544622; doi:10.1186/s12911-023-02276-3)

Supplementary material - Neurophysiologist questionnaire
MEP muscle identification – Train

There are four muscles: Thenar, Extensor Digitorum, Abductor Hallucis and Tibialis. The goal is to classify the MEP signals on the back. On this side, there are several MEP traces labelled by the corresponding muscle. You may look at these traces to «train», and then you should turn the page and try to classify the MEP traces on the back according to one of the four muscles.

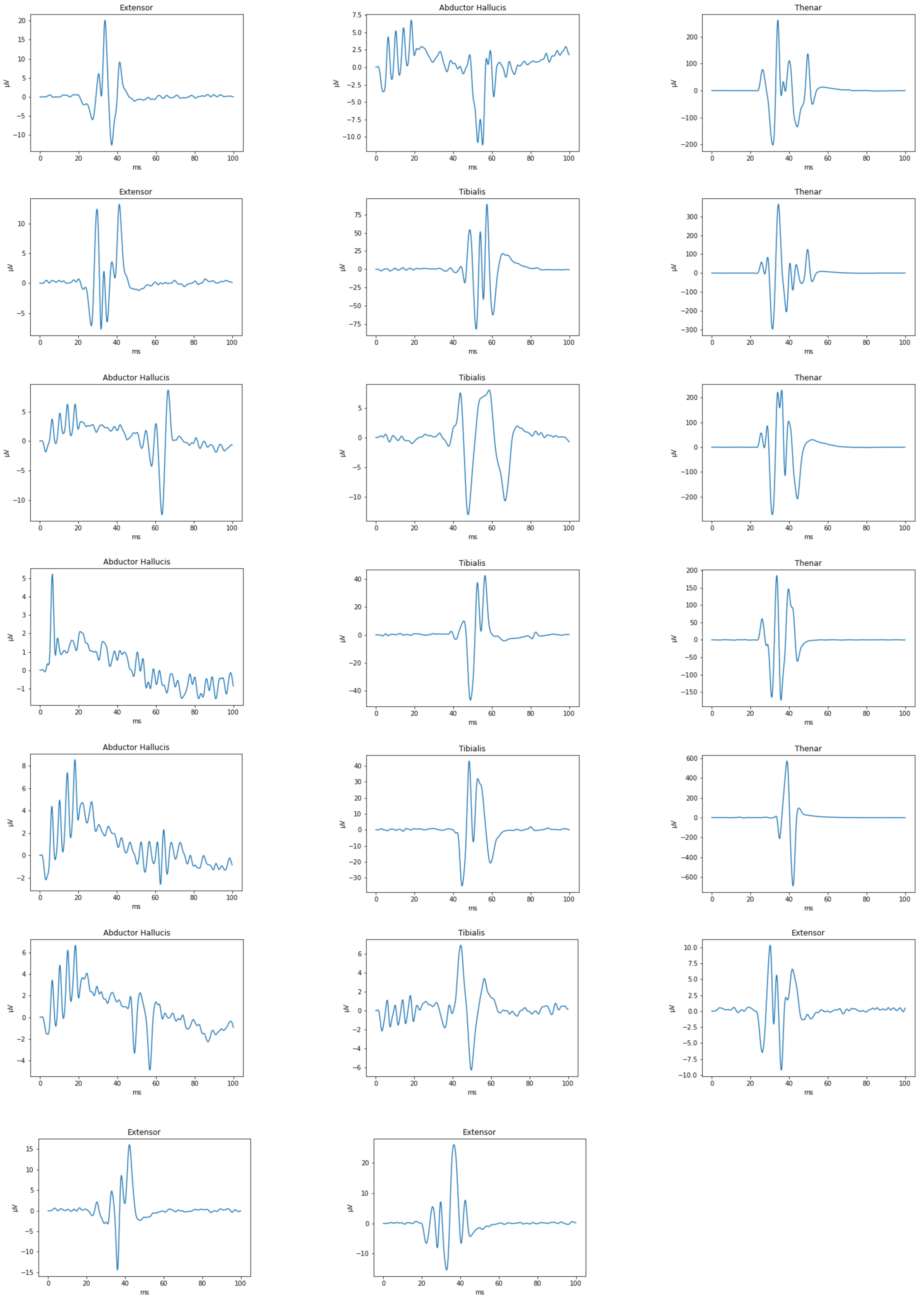

MEP muscle identification – Test

Please classify the following MEP traces according to the following four muscles: Thenar (APB), Extensor Digitorum (EXT), Abductor Hallucis (AH) and Tibialis (TA).

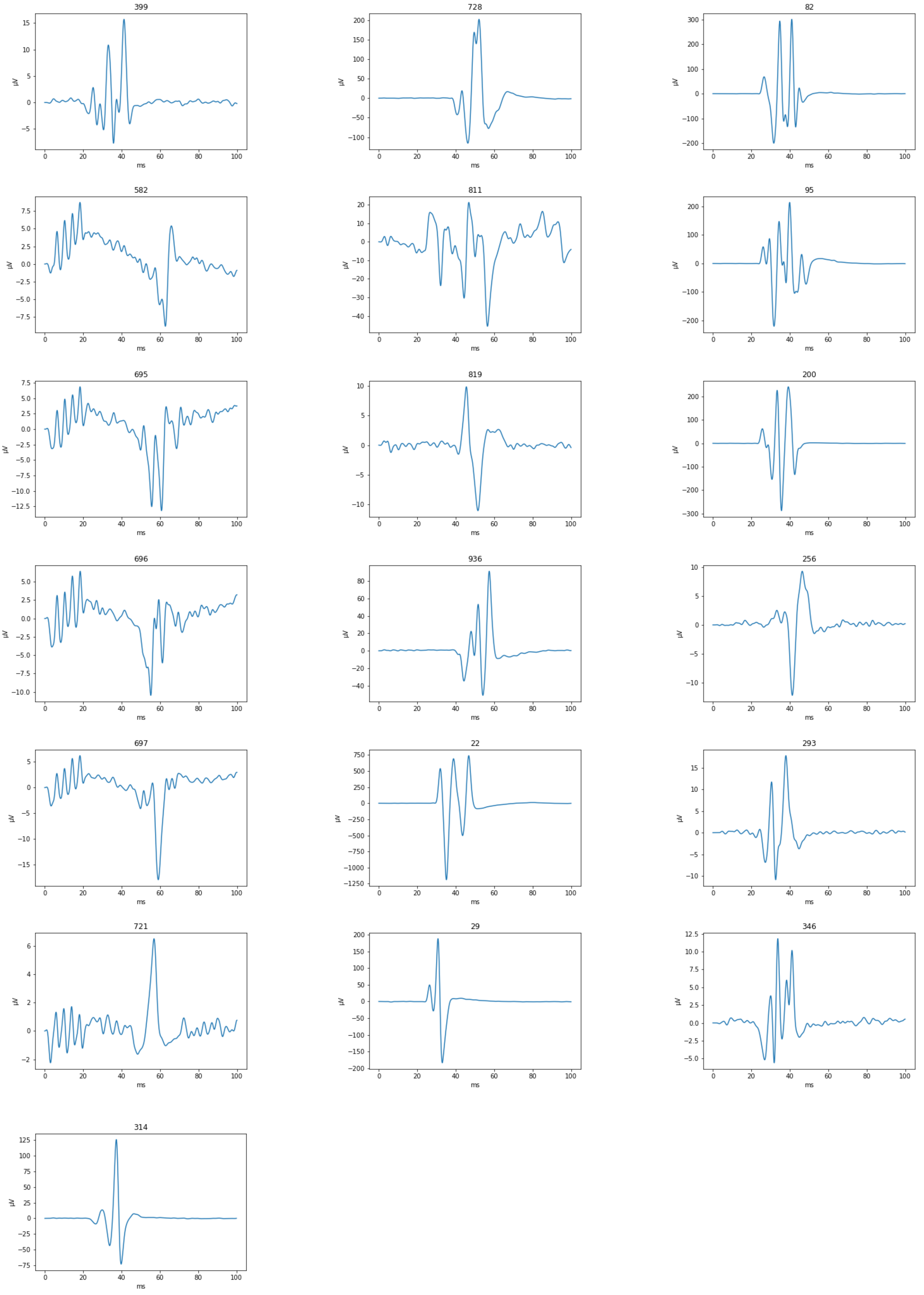

Supplement: Supplementary file 2 — Additional file 2. [file 12911_2023_2276_MOESM2_ESM.pdf]
